# The prognostic significance of micrometastases in node-negative squamous cell carcinoma of the vulva

**DOI:** 10.1038/sj.bjc.6602343

**Published:** 2005-01-18

**Authors:** G V Narayansingh, I D Miller, M Sharma, C J Welch, L Sharp, D E Parkin, M E Cruickshank

**Affiliations:** 1 Gynaecology Oncology Unit, Ward 43, Aberdeen Royal Infirmary, AB25 2ZN, UK; 2 Department of Pathology, Aberdeen Royal Infirmary, AB25 2ZN, UK; 3 Department of Epidemiology, University of Aberdeen, AB25 2ZN, UK

**Keywords:** vulval cancer, groin nodes, micrometastases

## Abstract

Nodal involvement is one of the most significant prognostic factors in squamous cell carcinoma (SCC) of the vulva. We conducted a retrospective analysis of 31 women with histologically node-negative SCC from a population-based cohort of Grampian women. Median follow-up was 42 months after radical vulvectomy with groin node dissection. In total, 13 women (42%) were found to have micrometastases on immunohistochemistry. The risk of recurrence was almost 20-fold higher in those with micrometastases compared to those without (hazard ratio=19.6 (95% CI 2.3–171).

Micrometastases, detected by imunohistochemistry (IHC) techniques, have been implicated as a causative factor for recurrence in node-negative patients with cancer. In breast cancer, for example, the presence of micrometastases in axillary nodes is an independent and a significant predictor of clinical outcome (Gray *et al*, 2001; Umekita *et al*, 2002). In cervical cancer, some preliminary work has detected micrometastases in histologically negative nodes (Van Trappen *et al*, 2001). They have been able to identify micrometastases using real-time PCR and relate this to cytokeratin 19 to establish a molecular quantification and mapping of lymph node micrometastases in cervical cancer. In terms of its clinical relevence, Juretzka *et al* (2004) noted that two out of four patients (50%) with micrometastases, who had previously histological negative lymph nodes in cervical cancer recurred in contrast to three out of 45 (7%) without micrometastases. Less is known about the relationship of micrometastases in the inguinofemoral nodes of patients with vulvar squamous cell carcinoma (SCC). Groin lymphadenectomy is an inherent part of the management of vulval cancer and women with histologically negative nodes are considered to be optimally managed and do not receive adjuvant therapy. Yet there is a 10–25% recurrence rate in this circumstance (Malfetano *et al*, 1985; Gordinier *et al*, 2003). Micrometastases may increase the risk of recurrence in vulval cancer patients, but this has not been clearly demonstrated to date. Our aim was to establish whether (1) micrometastases could be identified in histologically negative groin nodes and (2) whether there is an association between clinical recurrence and micrometastases.

## MATERIALS AND METHODS

**Figure 1:**
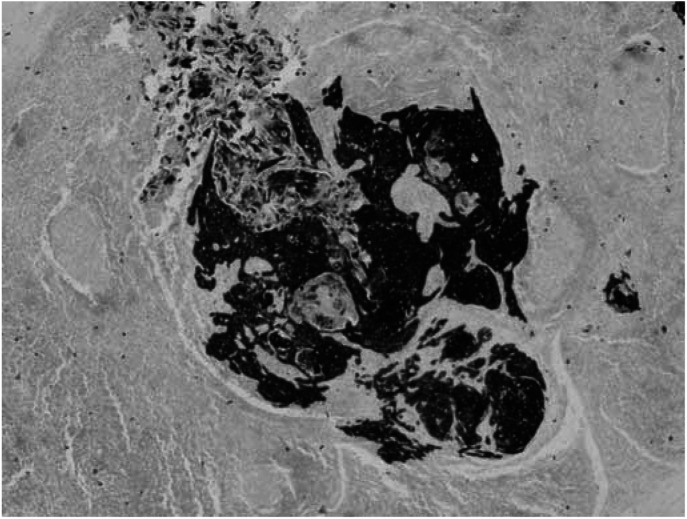
Micrometastases – cells in clusters.

All women in Grampian Region who had a groin dissection for vulval SCC with histologically negative nodes between 1988 and 1998 were identified from the Grampian Gynaecology Oncology database. None of these patients had adjuvant radiotherapy. Tumour blocks were identified from the pathology archive and the histology reviewed to confirm diagnosis, staging, tumour features and node negativity. All the lymph nodes were serially sectioned at intervals of 250 *μ*m (mean four levels per block). Four sections were made at each level, one stained with haematoxyllin and eosin (H&E), one stained with a broad-spectrum anticytokeratin monoclonal antibody (MNF 116) using immunohistochemistry (IHC) and one negative control for IHC with one spare. Although other molecular methods (Van Trappen *et al*, 2001) have been used to detect occult metastases in lymph nodes, we chose cytokeratin immunocytochemistry as this technique is relatively inexpensive and simple. In addition, the pathologists in this study are experienced in the interpretation of these preparations. Furthermore, from a laboratory standpoint, imunnocytochemistry is also more widely available compared to methods such as polymerase chain reaction. MNF116 was used as it demonstrated the most consistent, selective and intense staining of the malignant squamous cells and each of the lymph nodes was examined for the presence of micrometastases (see Figure 1).

General data with respect to the diameter of the primary, depth of invasion and the presence of coexisting lichen sclerosis or vaginal intraepithelial neoplasia was documented (Table 1). The site, number of cells and maximum diameter of the micrometastatic deposits were recorded. Dates of recurrence and death were obtained from the medical records. Data on the cause of death were not universally available. Women contributed follow-up time from the date of diagnosis with vulval SCC to date of first recurrence, date of death or end of follow-up (generally the date last seen on the clinic) whichever was the earliest. Cox proportional hazard methods were used to compare the risk of recurrence and the all-cause mortality in women with and without recurrence. Hazard ratio was adjusted for age at diagnosis.

## RESULTS

**Figure 2:**
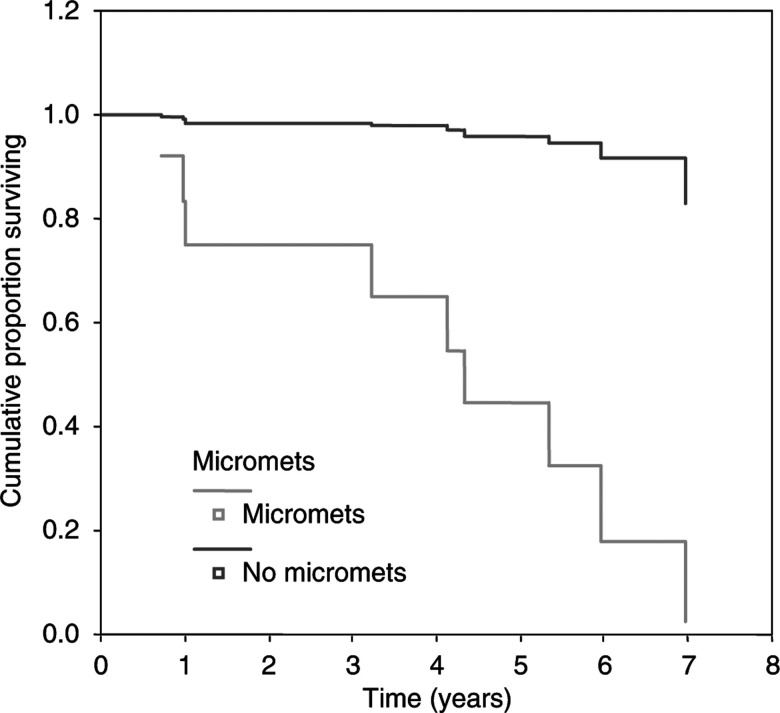
Relationship with time to recurrence and nodal status of micrometastases (adjusted for age).

In total, 43 cases were identified but 12 were subsequently excluded (case notes destroyed in eight, two cases no tumour, one case blocks missing and in one no nodes were removed) leaving 31 women in the study group. The median age at diagnosis was 70 years (range 30–94). The mean number of lymph nodes removed was 10 (range 2–19. The median duration of follow to recurrence was 39 months (range 1–102) and to death was 42 months (1–109). Nine women (29%) developed recurrent disease. On reanalysis with standard H&E, one case of nodal involvement was identified. After IHC staining, 13 (42%) of women were found to have micrometastases. Eight of the nine women who relapsed were in this group. Of the remaining 18 women without micrometastases, only one recurred. The age-adjusted hazard ratio for recurrence was 19.6 (CI 2.3–171) in those with micrometastases compared to those without (Figure 2). Six of the 13 patients (46%) with micrometastases had died compared to four of the 18 without micrometastases (22%). The age-adjusted hazard ratio for survival in those with micrometastases was 1.8 (CI 0.5–6.56), implying that the risk was slightly raised in those with micrometastases, but this did not reach significance and we were unable to look specifically at deaths due to vulval SCC.

## DISCUSSION

Our analysis demonstrates, firstly that micrometastases can be clearly identified in histologically node-negative patients with vulval cancer and, secondly and more importantly, that women with micrometastases are at a significantly increased risk of recurrence.

Two major issues of concern arising from the findings of this study are the drive towards less radical surgery to avoid the well-recognised morbidity associated with groin dissection and the additional laboratory workload required in ultrastaging of lymph nodes. There is considerable interest in examining the status of sentinel nodes in vulval cancer in an attempt to reduce the need for groin dissection. There has been a case reported of recurrence after sentinel node dissection where subsequent analysis confirmed that the negative sentinel node did contain micrometastases (Tamussino *et al*, 2002). Marchiole *et al* (2004) found that three out of five patients with nodal micrometastases in cervical cancer had tumour cells in nonsentinel nodes despite having negative findings. The high false negative rate with sentinel node biopsy in cervical cancer raises concerns about the accurate identification of sentinel nodes in this condition, which is in stark contrast to vulval cancer where the identification of sentinel nodes has been extensively studied with a higher predictive value. If less radical surgery for early stage vulval cancer is to be undertaken then there is justification for using serial sectioning and IHC staining in the sentinel node of the groin bed in order to obviate the likelihood of recurrent disease, as women with micrometastases may not receive optimal treatment.

Our 42% detection rate of micrometastases in previously node-negative patients is high compared to those in previous studies, which were generally in the range of 10–25% (Malfetano *et al*, 1985). This could be due to by the serial sectioning methodology with variable intervals between the levels examined as well as the sensitivity of the IHC antibody used. Routine histological examination in this historical cohort is likely to have been one 5-*μ*m-thick section and indeed one macrometastasis was missed. In our view, systematic serial sectioning and IHC is necessary to identify micrometastases. Interpretation of IHC in lymph nodes requires a degree of histopathological experience to discern true staining of the rounded metastatic carcinoma cells from anomalous staining due to the reaction products of the IHC and weak staining of interdigitating dendritic cells found in normal nodes. Serial sectioning and staining with IHC requires time and expertise and has cost implications. Further studies are necessary of larger cohorts to confirm our findings. Moreover, studies are needed to determine whether adjuvant therapy offers any advantage to women diagnosed with vulval SCC, who are node negative and have micrometatasses.

## Figures and Tables

**Table 1:** Patient's characteristics with respect to nodal micrometastatic status

		**No micrometastases, 18 patients**	**Micrometastases, 13 patients**	**Total, 31 patients**
Tumour diameter (cm)	0–2	10	6	16
	2.1–4	4	3	7
	>4.1	3	4	7
	Not recorded	1	0	1
				
Depth of invasion (mm)	<1	4	0	4
	1–10	12	10	22
	>10	1	3	4
	Not recorded	1	0	1
				
Associated benign vulval conditions	No lichen sclerosis or VIN	5	1	6
	Lichen sclerosis	4	11	15
	VIN	8	1	9
	Both lichen sclerosis and VIN	1	0	1
				
Recurrence		1	8	9
